# Sarcopenic Obesity, Insulin Resistance, and Their Implications in Cardiovascular and Metabolic Consequences

**DOI:** 10.3390/ijms21020494

**Published:** 2020-01-13

**Authors:** So-hyeon Hong, Kyung Mook Choi

**Affiliations:** Division of Endocrinology and Metabolism, Department of Internal Medicine, College of Medicine, Korea University, Seoul 08308, Korea

**Keywords:** sarcopenic obesity, insulin resistance, cardiometabolic disease

## Abstract

The prevalence of sarcopenic obesity is increasing worldwide, particularly amongst aging populations. Insulin resistance is the core mechanism of sarcopenic obesity and is also associated with variable cardiometabolic diseases such as cardiovascular disease, type 2 diabetes mellitus, and non-alcoholic fatty liver disease. Fat accumulation in muscle tissue promotes a proinflammatory cascade and oxidative stress, leading to mitochondrial dysfunction, impaired insulin signaling, and muscle atrophy. To compound the problem, decreased muscle mass aggravates insulin resistance. In addition, the crosstalk between myokines and adipokines leads to negative feedback, which in turn aggravates sarcopenic obesity and insulin resistance. In this review, we focus on the molecular mechanisms linking sarcopenic obesity and insulin resistance with various biological pathways. We also discuss the impact and mechanism of sarcopenic obesity and insulin resistance on cardiometabolic disease.

## 1. Introduction

Aging and obesity are the common public health issues worldwide. People older than 65 years comprise 13% of the global population, and this percentage is increasing at a more rapid rate compared to the percentage of any other age group [1]. With aging, the loss of muscle mass and strength occurs naturally, and is defined as primary sarcopenia. In addition, secondary sarcopenia can develop because of physical inactivity, malnutrition, and diseases, such as neurodegenerative disease, endocrine disease, or malignancy [2]. Reduced muscle mass synergistically accompanies accumulation of fat mass, resulting in sarcopenic obesity [3]. Compared to obesity alone, sarcopenic obesity is associated with a heightened risk of adverse health outcomes, such as disability or impairment, cardiometabolic diseases, other comorbidities, and mortality [4,5,6,7].

Cardiometabolic diseases, including cardiovascular diseases, type 2 diabetes mellitus, and non-alcoholic fatty liver disease (NAFLD), are the leading causes of death worldwide. Among several risk factors, obesity, excess calorie intake, and low levels of physical activity are the main contributors [8], and insulin resistance (IR) is a common mechanism associated with the disease [9]. IR is the core of the pathophysiological characteristics of sarcopenic obesity. Skeletal muscle is the largest insulin-sensitive tissue and has the largest requirement for postprandial glucose through insulin dependent mechanism. Hence, impaired insulin signaling is commonly observed in sarcopenic obesity [10,11].

In this review, we describe the molecular pathogenesis of sarcopenic obesity with a particular focus on IR. We discuss its roles in cardiometabolic diseases, including atherosclerosis, cardiovascular disease, chronic heart failure, type 2 diabetes mellitus, metabolic syndrome, and NAFLD.

## 2. Sarcopenia and Sarcopenic Obesity

### 2.1. Definition

Sarcopenia presents as a decline in skeletal muscle mass and strength. This decline is part of a normal physiological aging process; however, several factors exacerbate this situation, including low physical activity, inadequate nutrition, neurodegenerative disease, and inflammatory conditions. These factors result in increasing frailty and an increased risk of mortality [2,12]. Sarcopenic obesity, a combination of sarcopenia and obesity, is a concurrence of muscle loss and body fat increment. This body composition change brings unchanged or similar body weight or BMI; however, the change shifts toward unfavorable status, including reduced baseline metabolic rate, decreased mitochondrial number and volume, and increased oxidative stress, which exacerbates the vicious cycle [13]. This complex disorder results in a 2–3 times higher risk of functional disability than either sarcopenia or obesity alone [14].

The definition of sarcopenic obesity has not been universally established, and there are various diagnostic criteria for sarcopenia and obesity (Table 1). We previously reported that different definitions of sarcopenia resulted in differential impact on cardiometabolic risk factors. [15].

### 2.2. Molecular Mechanism of Sarcopenic Obesity

In sarcopenic obesity, muscle is present as type II muscle fiber atrophy and increased lipid deposition and adipocytes [13,22]. Fast type II muscle fibers switch to slow type I muscle fibers resulting in decreased muscle mass and strength [23]. A decrease in motor neurons and collagen deposition is also observed [24].

#### 2.2.1. Fat Accumulation

Intramyocellular lipid (IMCL) develops when the inflow of fatty acid exceeds the oxidative capacity of skeletal muscle [25]. IMCL predominantly comprises triacylglycerol; however, other lipid intermediates, such as diacylglycerol (DAG), long-chain acetyl coenzyme A, sterol esters, and sphingolipids, including ceramides, also exist [26,27]. These lipids activate PKC and phosphorylate the serine of insulin receptor substrate-1 (IRS-1), interfering with PI3K activation and blocking GLUT4 translocation [28]. GLUT4 is a membrane transporter that transports glucose from blood into myocytes and its dysfunction results in decreased glucose utilization and increased fatty acid oxidation in the mitochondria. Increased fatty acid inflow leads to an increase in the ATP/ADP ratio in the mitochondria, along with a reduced electron transfer chain. This then results in the inhibition of mitochondrial respiration, increase in ROS formation, myocyte toxicity, and finally, development of sarcopenia [29]. Intermyocellular adipose tissue (IMAT) and IMCL secrete myostatin, CCL2, TNF-α, IL-1β, and IL-6, thus inducing IR and lipotoxicity, which is discussed in the next section [30].

Peri-muscular adipose tissue (PMAT) is an ectopic fat deposition surrounding muscle. Increased PMAT enhanced nuclear translocation of the FoxO (forkhead box O) transcription factors and upregulates Atrogin1 and MuRF1, which leads to proteolysis in muscle tissues. The amount of PMAT is correlated with the severity of muscle atrophy [31].

The perilipin family of proteins (PLIN) is embedded in lipid droplets, and functions as a regulator of skeletal muscle lipid metabolism and mitochondrial oxidation [32,33]. PLIN comprises PLIN1 to PLIN5, and PLIN2 and PLIN 5 are predominantly found in muscle tissue [34,35]. Overexpression of PLIN5 in skeletal muscle increases the gene expression involving fatty acid oxidation mediating PPARα and PGC1α [33]. Ablation of PLIN5 results in reduced TAG storage, but increased sphingolipids such as ceramide and sphingomyelin, thus leading to IR in the skeletal muscle [36]. In cultured myocytes, a lipid droplet-associated protein perilipin 2 increases expression of NLRP3 inflammasome, resulting in impaired insulin-stimulated glucose uptake [37].

These results suggest that increased fat accumulation in muscle impairs energy metabolism as well as glucose homeostasis, leading to catabolic status and atrophy of the skeletal muscle.

#### 2.2.2. Inflammation

In sarcopenic obesity, adipocytes are accumulated not only in muscle tissue, but also in other organs. Increased adipose tissue secretes proinflammatory cytokines such as TNF-α, IL-6, and IL-1, and this promotes infiltration of inflammatory cells, including macrophages. In adipose tissue from lean subjects, approximately 10% of cells stained positive for macrophages, but in obese subjects, this number increased to up to 50% [38]. The infiltrated macrophages in muscle tissue present adipocyte-derived antigens and activate antigen-specific CD4^+^ T cells [39]. Macrophages also change the phenotype from M2 to M1, which releases proinflammatory molecules such as TNF-α, IL-1β, IL-6, and MCP-1/CCL2 [40,41]. These cytokines induce muscle atrophy by increasing apoptosis and upregulating proteosomal decay of filament proteins [42]. Using a proteomics approach, the cellular pathways activating macrophages are associated with sterile inflammation, which is distinct from classical activation by bacterial or viral infection [43,44]. In obesity-induced activation of macrophages, CD38, CD319, and CD274, are derived from the cell surface and only approximately 5% of the cell surface protein expression patterns overlap [43]. However, macrophages regulate inflammation in adipose tissue, and have autophagic functions whereby they prevent proinflammatory M1 macrophage-polarization by delivering cytoplasmic contents to lysosomes to degrade them and promote homeostasis through an mTOR activation process [44].

Taken together, increased fatty acid uptake and its accumulation in muscle induces inflammation with adipokines, proinflammatory cytokines, and other molecules, resulting in toxic effects on myocytes and ultimately sarcopenia. Sarcopenia results in a decreased metabolic rate, low physical activity, and myokine deficiency, which collectively leads to the proinflammatory effect, and exacerbates obesity.

#### 2.2.3. Sex-Specific Hormone and Growth Hormone Deficiency

Testosterone is an anabolic hormone that simulates protein synthesis by increasing amino acid utilization, and it also increases androgen receptor expression in skeletal muscle cells [45]. Consequently, testosterone increases muscle mass and insulin-like growth factor-1 (IGF-1) concentration and decreases inflammatory cytokines, such as IL-1 [46]. In obesity, the serum levels of testosterone decrease in response to increased aromatase activity, which converts testosterone to estradiol aromatization, resulting in hypogonadotropic hypogonadism [46]. Hypogonadal status also contributes to sarcopenic obesity by increasing levels of TNF-α and IL-6, which leads to central obesity [11,47].

In menopausal women, decreased levels of estrogen and increased levels of follicle-stimulating hormone and androgen resulted in increased deposition of visceral adipose tissues and decreased fat-free mass, leading to sarcopenic obesity [48].

Growth hormone (GH) is another anabolic hormone that activates multiple signaling cascades, and declines with aging and obesity. Low GH levels reduce IGF1 action by sequentially downregulating the PI3K-AKT/PKB-mTOR pathway, which induces protein synthesis in muscles [49]. The IGF-IR/IR knockout mice showed decreased skeletal muscle mass [50].

In brief, the process of sarcopenia, due specifically to decreased production of sex-specific hormone and growth hormone with aging, is aggravated by obesity.

## 3. Sarcopenic Obesity and Insulin Resistance

Skeletal muscle is the largest major organ for disposal (or reservoir) of postprandial glucose ingestion via an insulin dependent mechanism [11,51]. IR reflects the status of impaired insulin signaling, which consequently results in glucose transport and metabolism process that are closely related to sarcopenic obesity.

### 3.1. At Skeletal Muscle

In the normal physiology of skeletal muscle, insulin binds to the transmembrane insulin receptor, autophosphorylates tyrosine residues in the activation loop, activates IRS-1 and IRS-2, and successively activates PIC3K. PIC3K phosphorylates membrane lipid PIP2, converting it to PIP3. PIP3 binds to Akt, which catalyzes the phosphorylation of proteins, facilitating glycogen synthesis and translocation of GLUT4. GLUT4 moves through the plasma membrane and then acts in a dose-dependent manner. Imported glucose is stored as glycogen or enters the glycolytic pathway, and is either oxidized or released as lactate [52]. Additionally, insulin is involved in the maintenance of muscle mass via the p38MAPK and the mTOR/p70S6 kinase pathway, thus suppressing proteolysis [53,54,55].

In sarcopenic obesity, the increased lipid metabolites, such as including DAG and ceramides, activate serine/threonine kinases such as PKC, C-Jun NH2-terminal kinase (JNK), and IKK, resulting in the phosphorylation of serine/threonine in the insulin receptor protein and its substrates, resulting in impaired insulin signaling [28,56]. In lipid infusion experiments, DAG content in the muscle increases, and consequently PKCθ is activated, resulting in limited phosphorylation of IRS-1 and glucose uptake through GLUT4 [9]. PKCθ impairs muscle insulin signaling, inhibiting insulin-mediated glucose uptake through GLUT4.

IR correlates with impaired lipid oxidation in the mitochondria [57]. The accumulation of lipids and their metabolites, particularly long chain fatty acids, causes lipotoxicity, and promotes ROS production and ER stress as a result of inflammation, which in turn leads to impaired mitochondrial dysfunction [57]. Mitochondrial dysfunction results in further intracellular fatty acid ambulation, creating a vicious cycle of lipotoxicity [58]. An increase in oxidant compounds activates the stress pathways including IKK, JNK, and p38-MAPK [59].

While a report shows that local inflammation induced by muscle MCP-1 overexpression interferes with insulin signaling in muscle [60], the other study does not report a similar observation [61]. The following signaling pathways are currently being examined for sarcopenic obesity and IR.

#### 3.1.1. IKK/NF-kB Pathway

The IKK/NF-kB pathway is activated by TNF-α, IL-1β, and free-fatty acids [62]. IKK-mediated serine phosphorylation of IRS-1 or insulin receptor results in impairment of insulin-induced tyrosine phosphorylation. It results in decreased GLUT4 expression and increased nitric oxide synthase (iNOS) production, leading to IR [63,64,65].

#### 3.1.2. MAPK Family

JNK is a member of the MAPK family and is activated by TNF-α, IL-1β, free-fatty acids, ER stress, and LTB4 [66,67]. JNK causes IR by inducing serine and threonine phosphorylation of IRS and attenuating downstream insulin signaling [68]. Activated p38 MAPK is also involved in muscle IR [69]. Palmitate-induced IR in myocytes shows increase in JNK activity and knockdown of JNK in mice, which shows alleviated IR by enhanced fatty acid utilization and glucose oxidation [70,71].

#### 3.1.3. JAK/STAT

IFN-γ activates JAK1 and JAK2 inducing STAT1 phosphorylation. IL-6 and palmitate causes STAT3 phosphorylation. It is not fully understood how the JAK-STAT pathway leads to IR in muscle tissues, and controversial results from previous studies have suggested that muscle-specific STAT3 in mice does not alter IR [72,73].

The suppressor of cytokine signaling (SOCS), particularly SOCS1 and SOCS3, are proteins inhibiting inflammatory signaling by blocking JAK activity [74]. This suppressing activity interferes with the crosstalk between the insulin receptor and IRS by inhibiting insulin receptor tyrosine kinase activity and degrading IRS [74,75]. In mice, macrophage-specific ablation of SOCS1 increases systemic inflammation and IR [76], and a loss of function of the cavin-1 binding sites on SOCS3 consequently induces lipodystrophy and IR in mice [77].

#### 3.1.4. PKCs

PKC is a family of serine/threonine kinase activated by phospholipase C and hydrolysis of membrane phosphoinositides [78]. PKCs are grouped into three classes, as follows: classical PKCs (α, β1, β2, γ), novel PKCs (δ, ε, η, θ), and atypical PKCs (ζ, λ/ ι) [79]. PKCθ is predominantly expressed in skeletal muscle and endothelium and is activated by increased DAG content in muscles, via elevated plasma fatty acid infusion in humans [80] or palmitate treatment in myocytes [64]. Similar to IKK and JNK pathways, it induces serine or threonine phosphorylation or IR or IRS-1, resulting in impairment of insulin signaling [80,81].

### 3.2. Crosstalk Between Myocyte and Adipocytes

#### 3.2.1. IL-6

IL-6 is a cytokine produced by numerous cell types, such as myocytes, adipocytes, and leukocytes, and it has dual effects in inflammation [82]. As a myokine, IL-6 releases from the skeletal muscle in condition of acute muscle contraction without damage [83]. Acute increment of IL-6 in healthy humans in association with classic signaling, improved insulin sensitivity by enhancing glucose uptake, and increased fatty acid oxidation in myocytes [84]. In the pancreas, IL-6 stimulates insulin secretion by beta cells by upregulating glucagon-like peptide-1 (GLP-1) [85]. It also induces anti-inflammatory cytokines, such as IL-10, and inhibits the feedback of the TNF-α pathway [59].

In contrast, in chronic states such as obesity, IL-6 acts as a pro-inflammatory cytokine via the trans-signaling pathway. A high-fat diet leads to chronically elevated levels of IL-6, which then recruits macrophages in the adipose tissue [86]. This results in decreased IGF-1 levels and reduced muscle mass and strength by elevated expression of SOCS3 [87,88]. Chronically elevated IL-6 levels increase MAP3K8 expression in adipose tissue, which is a signal transducer that regulates activation of NF-kB and JNK transcription factors and leads to IR [89]. Long-term increment of serum IL-6 levels prospectively predicts unstable atherosclerotic plaques in patients with internal carotid artery stenosis [90]. In older, healthy subjects, progressive strength training reduced serum IL-6 levels compared to that in a control group [91].

Therefore, IL-6 is not only involved in the communication between skeletal muscle and adipose tissue, but also involved in the crosstalk between the pancreas and liver to control glucose metabolism and the cardiovascular system, which, in turn, may control sarcopenia and obesity.

#### 3.2.2. Irisin

Irisin is a PPARγ-coactivator-1α (PGCα)-dependent myokine that stimulates browning in subcutaneous adipose tissues and induces increased energy expenditure, which improves obesity and IR through the MAPK and ERK pathways [92,93]. Irisin also stimulates beta-cell survival and glucose-stimulated insulin secretion through the PGA pathway, and inhibits saturated fatty acid-induced apoptosis in pancreatic beta-cells [94]. A low level of irisin in mice with muscle-specific constitutive ROCK1 activation suppresses irisin production in muscles, decreasing browning in adipocytes and leading to impaired insulin sensitivity [95].

In human studies, the level of plasma irisin was positively associated with muscle mass and strength and negatively associated with the fasting glucose level [96]. However, the results on the association between exercise and change in irisin expression are conflicting because of differences among studies, in the exercise regimen programs prescribes, the characteristic of the study populations, and the types of measurement assays used [97,98,99].

Taken together, irisin has a beneficial effect on metabolic homeostasis, but further investigations are needed to find the role of irisin in sarcopenic obesity and cardiometabolic diseases.

#### 3.2.3. Adiponectin

Adiponectin is one of the adipokines that is released from adipocytes, and regulates appetite and energy expenditure. In obesity, the serum level of adiponectin decreases. Thus, it facilitates insulin sensitivity by increasing glucose uptake in skeletal muscle and by stimulating fatty acid oxidation through activation of the 5′-AMP-activated protein kinase (AMPK) signaling pathway [100]. Adiponectin also decreases inflammation by inhibiting TNF-α and IL-γ secretion and elevating IL-10 and IL-1 receptor antagonist production from monocytes and macrophages [82]. In contrast, TNF-α impairs adiponectin signaling, mitochondrial biogenesis, and oxidative capacity, resulting in impaired functionality of skeletal muscles [101].

In human studies, resistance and aerobic exercise improved sarcopenic obesity and increased the adiponectin levels in overweight or obese breast cancer survivors [102]. However, there is a controversy about whether adiponectin reflects skeletal muscle improved by exercise or fat accumulation. In overweight or obese postmenopausal women, the adiponectin level increased by 9.5% in the diet intervention group but by 6.5% in both diet and exercise groups [103].

In summary, adiponectin is negatively associated with obesity and has a protective effect for inflammation and lipid accumulation in skeletal muscle, but further studies are needed to elucidate its clinical effect, particularly in the subjects with sarcopenic obesity.

## 4. Pathophysiological Link among Cardiometabolic Disease, Sarcopenic Obesity, and Insulin Resistance

### 4.1. Atherosclerosis and Cardiovascular Disease

Atherogenesis is a complicated process that includes oxidative stress, inflammation, endothelial dysfunction, vascular proliferation, and thrombosis [104]. Atherosclerotic plaque rupture can lead to myocardial infarction and stroke [105]. Hyperinsulinemia due to IR impairs the IRS-1/PI3K/Akt pathway, which causes vasodilation by activating nitric oxide synthase (NOS) and leads to the production of nitric oxide (NO) [106]. This results in impaired vasodilation and endothelial dysfunction, leading to the development of atherosclerosis. In contrast, as the MAPK pathway has normal sensitivity to insulin, hyperinsulinemia activates the MAPK pathway, promoting vascular smooth muscle cell growth and activating inflammatory pathways, such as IkB/NF-kB and JNK [107,108].

In adipocytes, IR by impaired insulin/mTOR2 signaling results in increased MCP1 production, which results in increased circulating MCP1. This activates the proinflammatory M1 macrophages which release cytokines such as TNF-α [109]. When macrophages are under oxidative stress, oxidized lipids such as 7-hydroperoxide proceed to inactivate CYP27A1, reducing ABCA1/G1 expression and ultimately leading to impaired cholesterol efflux to the extracellular apoA-I or HDL [110]. Excessive oxidized LDL cholesterol imported into macrophages leads to the development of foam cells that are deposited beneath the endothelium in arteries, forming atherosclerotic plaques [110].

In a clinical study, patients with sarcopenic obesity had higher carotid intima-media thickness (IMT) and oxidative stress markers compared to sarcopenic non-obese and/or non-sarcopenic patients [111]. Among patients with myocardial infarction, those with visceral obesity had elevated levels of proinflammatory cytokines, such as TNF-α, IL-1b, IL-6, IL-8, IL-12, and CRP than those without visceral obesity [112]. A British regional health study reported that older subjects with sarcopenic obesity had the highest cardiovascular mortality [113].

These results suggest that sarcopenic obesity mediated IR accompanies vascular smooth muscle growth and vascular endothelial damage by chronic inflammation and oxidative stress, leading to atherosclerosis and cardiovascular disease [114].

### 4.2. Chronic Heart Failure

Chronic heart failure is the status of a clinical endpoint with decreased pumping function, resulting in impaired organ perfusion usually followed by myocardial infarction or stroke. IR and chronic inflammation are observed in chronic heart failure. Sarcopenic obesity shares these conditions as well.

Hyperinsulinemia, due to IR, activates the renin-angiotensin-aldosterone system (RAS). Increased levels of angiotensin II and following increased aldosterone levels activates the angiotensin receptor 1 and mineralocorticoid receptors, respectively, which stimulates mTOR-S6K1 signaling in heart IR [115,116]. The mTOR-S6K1 pathway increases the expression of epithelial sodium channels in the vascular cell membrane and activates serum-and-glucocorticoid-regulated kinase 1, which leads to reduced NO production and increased cardiac stiffness [117]. Moreover, angiotensin II type 1 receptor and mineralocorticoid receptor activate the TGF-β1-SMAD pathway, which increases myocardial fibrosis and structural remodeling [118]. Increased RAS also activates sympathetic neurons that contribute to impaired autonomic regulation in heart failure. RAS activity in brain paraventricular nucleus upregulates MAPK signaling and results in sympathetic excitation in rats with heart failure [119].

Inflammation due to sarcopenic obesity results in reduced NO, cyclic guanosine monophosphate (cGMP), and protein kinase G (PKG) activity in cardiomyocytes, leading to LV hypertrophy and stiffness [120]. Activated macrophages release TNF-α, IL-1b, IL-6, and TGF-β, which stimulate myofibroblasts, increase collagen production, and decrease protease inhibitor secretion [121,122]. As a result, the extracellular matrix is degraded by Smad-dependent and/or independent signaling pathways and fibrosis occurs in both cardiac and skeletal muscles [123].

Reduced mitochondrial function leads to impaired muscle contractile function and cardiomyocyte death. In rats with heart failure, pro-oxidants such as NADPH oxidase and XO activity were higher, and antioxidants, such as catalase and superoxide dismutase (SOD), were lower than in corresponding controls [124].

In a clinical study, patients with sarcopenia and/or obesity had a higher risk of co-morbidity and poor prognosis with chronic heart failure [125]. Even in subjects without heart failure, myocardial infarction, and diabetes mellitus, fasting plasma insulin level was positively associated with adverse echocardiographic features, which predispose them to a risk of heart failure [126]. Furthermore, patients with chronic heart failure showed adiponectin resistance in myocardial and skeletal muscle cells through increased oxidative stress [127].

### 4.3. Type 2 Diabetes Mellitus

IR plays a major role in the pathogenesis of type 2 diabetes mellitus [128]. Even though pancreatic beta cell dysfunction is the main pathogenesis of type 2 diabetes mellitus, given that the uptake of postprandial glucose by skeletal muscles is approximately 80–90%, skeletal muscle IR plays a crucial role in the development and progression of diabetes mellitus [129,130].

In a clinical study, patients with sarcopenic obesity with higher BMI and lower hand grip strength had a higher risk of type 2 diabetes mellitus than controls did [131]. We also previously reported that patients with type 2 diabetes mellitus had higher BMI and a low appendicular skeletal mass and skeletal muscle index [132].

### 4.4. Metabolic Syndrome

Metabolic syndrome shares risk factors for type 2 diabetes mellitus and atherosclerotic cardiovascular disease, which tend to namely be abdominal obesity, hyperglycemia, dyslipidemia, and hypertension. Another name for the syndrome is IR syndrome. It is closely associated with IR and obesity, and excess energy intake is usually the underlying cause of the syndrome [133]. Fat accumulation increases oxidative stress due to increased ROS production and NADPH oxidase activation [134]. It causes the dysregulation of adipocytokines, such as adiponectin, PAI-1, IL-6, and MCP-1, leading to metabolic syndrome [134]. IR also activates SREBP-1c and inhibits acetyl-coenzyme A carboxylase, leading to reduced PPAR-a expression and the promotion of large-sized and TG-rich VLDL synthesis and secretion [135].

Most clinical studies have reported that sarcopenic obesity is associated with metabolic syndrome [136,137,138]. However, in an Australian study assessing older men, sarcopenic obesity was associated with metabolic syndrome; however, obesity itself, rather than the sarcopenia measured by ALM, grip strength, or gait speed was more predictive of metabolic syndrome [139].

### 4.5. NAFLD

IR is the main characteristic of NAFLD and is observed in the liver, skeletal muscle, and adipose tissue in sarcopenic obesity [140]. Sarcopenic obesity seems to impair insulin signaling in hepatocytes, resulting in increased de novo hepatic lipogenesis and inhibited hepatic β-oxidation [141]. Moreover, obesity accompanies the accumulation of lipids in the liver, thus aggravating insulin resistance in hepatocytes [141]. In genetic mouse models of muscle-specific IR, by deleting the IRTK [142] or GLUT4 [143] genes, hepatic steatosis and adiposity were increased [142].

Chronic inflammation releases cytokines TNF-α, IL-6, and CRP in muscle and adipose tissues, aggravating IR and hepatic injury [144]. Decreased adiponectin levels are also noted in advanced hepatic injury [145]. When hepatocyte injury occurs, oxidative stress activates Kupffer cells, which release TNF-α and IL-6, leading to chronic inflammation and liver fibrogenesis progression [146]. Recently, it was reported that NAFLD affects intestinal microbiota, resulting in decreased immune T-cells and increased proinflammatory markers, including TNF-α, IL-6, and IFN-γ [147].

In a longitudinal cohort, subjects with sarcopenia were shown to have a higher risk of NAFLD and increased skeletal muscle index resolute [148]. Several cross-sectional studies have shown an association between sarcopenia and/or obesity and NAFD [144,149]. We have also reported that sarcopenia is independently associated with IR, as measured by HOMA-IR and NAFLD [150].

## 5. Conclusions

Accumulating evidence indicates that cardiometabolic diseases are associated with sarcopenic obesity and IR, and both play pivotal roles in disease development and progression. The clinical definition of sarcopenic obesity has not been unified and each set of diagnostic criteria has different clinical implications. However, the defining phenomena include decreased muscle mass and reduced strength of the muscle irrespective of size, and increased adiposity. Sarcopenic obesity is associated with (i) inflammation cascades that release proinflammatory cytokines, including TNF-α, IL-1, and IL-6, and activate M1 macrophages, and (ii) alterations in growth hormone, testosterone, and estradiol. In skeletal muscles, these changes activate several pathways, such as the IKK/NF-kB, JNK, PKC, and JAK/STAT pathways, which result in IR. In sarcopenic obesity accompanied IR, the proposed mechanisms lead to multiple cardiometabolic diseases, including cardiovascular disease, atherosclerosis, chronic heart failure, type 2 diabetes mellitus, metabolic syndrome, and NAFLD. Further studies are needed to clarify the mechanisms contributing to the conflicting molecular and clinical results that are evident between cardiometabolic diseases and sarcopenic obesity with IR. Advancing the understanding of sarcopenic obesity and IR may lead to the emergence of novel therapies to prevent cardiometabolic diseases in aging populations.

## Figures and Tables

**Table 1:** Diagnostic criteria for sarcopenic obesity.

Study	Definition of Sarcopenia	Definition of Obesity
EWGSOP2 [2] Use the SARC-F questionnaire to find subjects with sarcopenia	Decreased muscle mass and Decreased muscle strength or performance	NA
	Muscle mass measurement ASM < 20 kg (M), 15 kg (W) ASM/height^2^ < 7.0 kg/m^2^ (M), < 6.0 kg/m^2^ (W) (DXA) Muscle strength measurement Hand grip strength < 27 kg (M), <16 kg (W) Chair stand > 15 s for five rises Performance measurement Gait speed ≤ 0.8 m/s SPPB ≤ 8 TUG ≥ 20 s 400 m walk test Non-completion or ≥6 min for completion	
New Mexico Aging Process Study [16]	ASM/height^2^ < 7.26 kg/m^2^ (M), <5.45 kg/m^2^ (W) (DXA)	Body fat > 27% (M), >38% (W)
NHANES III [17]	ALM/height^2^ < 9.12 kg/m^2^ (M), <6.53 kg/m^2^ (W)	Body fat > 27% (M), >38% (W)
FNIH [18]	ALM < 19.75 kg (M), <15.02kg (W) (DXA)	NA
Asian Working Group for Sarcopenia [19]	Decreased muscle mass and Decreased muscle strength or performance	NA
	Muscle mass measurement ALM/height^2^ < 7.0 kg/m^2^ (M), <5.4 kg/m^2^ (W) (DXA) ALM/height^2^ < 7.0 kg/m^2^ (M), <5.7 kg/m^2^ (W) (BIA) Muscle strength measurements Hand grip strength < 26 kg (M), <18 kg (W) Performance measurement Gait speed ≤ 0.8 m/s	
Korea Sarcopenic Obesity Study [20]	SMI < 7.26 kg/m^2^ (M), < 5.45 kg/m^2^ (W) (DXA)	Body fat > 27% (M), >38% (W)

SARC-F is a five-question self-reported questionnaire to screen for sarcopenia risk. The question comprises the subjects’ perception of his/ her limitations in strength, walking ability, rising from a chair, stair climbing, and experiences with falls [21]. ALM; appendicular lean mass. ASM; appendicular skeletal muscle mass. SPPB; Short physical performance battery. TUG; Timed-Up and Go test. SMI; Skeletal muscle index (total skeletal muscle mass (kg)/height (m)^2^). VFA; Visceral fat area.
